# Emerin anchors Msx1 and its protein partners at the nuclear periphery to inhibit myogenesis

**DOI:** 10.1186/s13578-019-0296-9

**Published:** 2019-04-11

**Authors:** Zhangjing Ma, Huiyuan Shi, Yi Shen, Huixia Li, Yu Yang, Jiange Yang, Hui Zhao, Gang Wang, Jingqiang Wang

**Affiliations:** 1 State Key Laboratory of Genetic Engineering and Collaborative Innovation Center of Genetics and Development, School of Life Sciences and Zhongshan Hospital, Fudan University, Shanghai, 200438 People’s Republic of China; 2 Zhengzhou Revogene Inc, Zhengzhou, 450000 People’s Republic of China; 3 State Key Laboratory of Cell Biology, CAS Center for Excellence in Molecular Cell Science, Institute of Biochemistry and Cell Biology, Shanghai Institutes for Biological Sciences, Chinese Academy of Sciences, Shanghai, 200031 People’s Republic of China

**Keywords:** Msx1, Emerin, Ezh2, H3K27me3, Nuclear-periphery, Myogenesis

## Abstract

**Background:**

Previous studies have shown that in myogenic precursors, the homeoprotein Msx1 and its protein partners, histone methyltransferases and repressive histone marks, tend to be enriched on target myogenic regulatory genes at the nuclear periphery. The nuclear periphery localization of Msx1 and its protein partners is required for Msx1’s function of preventing myogenic precursors from pre-maturation through repressing target myogenic regulatory genes. However, the mechanisms underlying the maintenance of Msx1 and its protein partners’ nuclear periphery localization are unknown.

**Results:**

We show that an inner nuclear membrane protein, Emerin, performs as an anchor settled at the inner nuclear membrane to keep Msx1 and its protein partners Ezh2, H3K27me3 enriching at the nuclear periphery, and participates in inhibition of myogenesis mediated by Msx1. Msx1 interacts with Emerin both in C2C12 myoblasts and mouse developing limbs, which is the prerequisite for Emerin mediating the precise location of Msx1, Ezh2, and H3K27me3. The deficiency of Emerin in C2C12 myoblasts disturbs the nuclear periphery localization of Msx1, Ezh2, and H3K27me3, directly indicating Emerin functioning as an anchor. Furthermore, Emerin cooperates with Msx1 to repress target myogenic regulatory genes, and assists Msx1 with inhibition of myogenesis.

**Conclusions:**

Emerin cooperates with Msx1 to inhibit myogenesis through maintaining the nuclear periphery localization of Msx1 and Msx1’s protein partners.

## Background

Dynamic and well-organized nuclear organization guarantees precise gene expression pattern in life activities. Genes acquire distinct expression states in different biological contexts, either being activated or repressed. The localization of genes changes according to expression states. Under most circumstances, activated genes tend to be distributed in the interior nucleus, while repressed genes are localized closely to the nuclear periphery [1–4]. Several factors participate in the regulation of repressed genes’ subnuclear localization, such as inner nuclear membrane proteins, non-coding RNAs, transcriptional factors, and histone deacetylases [5–8]. Among these factors, inner nuclear membrane proteins contribute to a stable repressive environment for genes supposed to be repressed, and maintain heterochromatin architecture [7].

One of the representative inner nuclear membrane proteins is Emerin, whose mutation or deletion leads to X-Emery-Dreifuss muscular dystrophy (X-EMED) [9, 10]. Emerin locates at the inner nuclear membrane of skeletal muscle cells, cardiac muscle cells, and smooth muscle cells [11–13], and belongs to LEM domain proteins [11, 14]. LEM domain mediates anchoring of chromosomes to the nuclear membrane and facilitates formation of higher-order chromatin structure through interacting with barrier-to-auto-integration factor (BAF) [15–19], a highly conserved protein capable of regulating higher-order chromatin structure [20], nuclear assembly [20, 21], and target genes’ expression [22, 23]. Like most other LEM domain proteins, Emerin is closely related to the structure of the nuclear membrane and heterochromatin [12, 24]. For instance, Lamin A, Lamin C and Lamin B2 are prone to be dissolved in skin fibroblasts of patients who are lack of Emerin, suggesting the destruction of lamina structure in these Emerin deficient cells [25]. The myoblasts of Emerin mutated patients are abnormal in heterochromatin architecture [26]. What’s more, Emerin is qualified to regulate genes’ nuclear position under certain conditions. Demmerle and collaborators showed that Emerin is able to cooperate with histone deacetylase 3 (HDAC3) to control the nuclear positions of some genes encoding myogenic transcriptional factors during myogenesis [27].

Msx1 is a homeoprotein specifically expressed in proliferating cells during skeletal muscle development and regeneration [28–30]. In myogenic lineage, the expression of Msx1 is restricted to myogenic precursors and activated satellite cells, both of which retain an undifferentiated state [29, 30]. Overexpression of Msx1 in C2C12 myoblasts inhibits the cells’ terminal differentiation to myotubes [31]. Several studies have shown that Msx1 mediates inhibition of myoblasts’ differentiation as a transcriptional repressor through down-regulating major myogenic differentiation regulators [32–35]. For instance, Msx1 binds to the core enhancer region (CER) of *MyoD* under the help of linker histone H1b to inhibit the transcription of *MyoD* in vitro and in vivo [32]. Apart from canonical transcriptional regulation, Msx1 inhibits the expression of several myogenic differentiation regulators on epigenetics level [33–35]. Our previous works found that in myogenic precursors, the repressed target myogenic regulatory genes of Msx1 are localized at the nuclear periphery [33–35]. Msx1 redistributes repressive histone marks H3K9me2 and H3K27me3 through recruiting corresponding histone methyltransferases to target genes at the nuclear periphery to keep the chromosomes in a repressive state [33–35]. The recruitment of histone methyltransferases to the nuclear periphery and the redistribution of repressive histone marks are required for Msx1 to keep myogenic precursors in an undifferentiated state [33–35]. However, the mechanisms by which Msx1, histone methyltransferases, and repressive histone marks co-localize at the nuclear periphery during inhibition of myogenic differentiation still remain elusive.

Here we show that Emerin, an inner nuclear membrane protein, is indispensable for the nuclear periphery localization of Msx1, histone methyltransferase Ezh2, and repressive histone mark H3K27me3 in C2C12 myoblasts. We identified Emerin as a protein interacted with Msx1 by immunoprecipitation coupled with Mass Spectrometry (IP-MS), and further validated their interaction in vitro and in vivo through co-immunoprecipitation (Co-IP) in cell lines and mouse developing limbs respectively. We found that the distribution of exogenous Msx1, endogenous Ezh2, and repressive histone mark H3K27me3 was altered from the nuclear periphery to interior nucleus in Emerin deficient cells, indicating the role of Emerin in mediating the localization of Msx1 and its protein partners. Furthermore, the expression levels of Msx1’s repressive genes were up-regulated in Emerin deficient cells compared with control cells when Msx1 was overexpressed in C2C12 myoblasts. Cells without Emerin were partially differentiated even with exogenous Msx1. Taken together, these observations provide a nuclear periphery anchoring model describing the relationship among Emerin, Msx1, and Msx1’s protein partners, in myogenesis.

## Materials and methods

### Description of plasmids

All plasmids used in this study have been described previously [31–33, 35, 36].

### Cell culture analyses

Cell culture studies were done using human 293T cells or retrovirus packaging Phoenix E cells or mouse C2C12 myoblasts obtained from ATCC. All cells were maintained in Dulbecco’s Modified Eagle Medium (DMEM) (Gibco) supplemented with 10% fetal bovine serum (FBS) (Gibco, Australian origin) in humidified atmosphere with 5% CO_2_ at 37 °C. Myogenesis of C2C12 myoblasts was induced by DMEM supplemented with 2% horse serum (Gibco) for 3–5 days. Lipofectamine 2000 reagent (Invitrogen) was used for transient transfection. Transient transfection was performed when cell confluence was over 70% according to the manufacturer’s recommendations. For exogenous genes delivered by retrovirus infection, replication-defective retroviruses were packaged using Phoenix E cells by transfection of the relevant pLZRS-IRES-GFP plasmid derivatives using Lipofectamine 2000 reagent (Invitrogen). C2C12 myoblasts were seeded at low density (lower than 10%) 12–24 h before infection with viral supernatants for 2 consecutive days. For siRNA transfection, C2C12 myoblasts were firstly infected with the viruses expressing Msx1 or the empty vector, then transfected with siRNA against Emerin using the Lipofectamine RNAiMAX reagent (Invitrogen) according to the manufacturer’s recommendations. Sequences of siRNAs against Emerin were shown below. Sense Emerin#1: 5′-GGGCUUAUCAUAUUAUCCU-3′; Antisense Emerin#1: 5′-AGGAUAAUAUGAUAAGCCC-3′. Sense Emerin#2: 5′-GCAAGGACUAUAAUGAUGA-3′; Antisense Emerin#2: 5′-UCAUCAUUAUAGUCCUUGC-3′. Sense Emerin#3: 5′-GACCUCACUUGUAGAUGCU-3′; Antisense Emerin#3: 5′-AGCAUCUACAAGUGAGGUC-3′.

### Animal studies

The animals were handled and cared for in accordance with the guidelines of the Animal Ethics Committee of Fudan University. Developing limb buds of wild-type mouse embryos (E11.5) were used to perform co-immunoprecipitation (Co-IP) assays.

### Immunoprecipitation coupled with Mass Spectrometry (IP-MS) analysis

Nuclear extracts from C2C12 myoblasts expressing Flag-Msx1 or Myc-Msx1 were immunoprecipitated with Anti-Flag/Anti-c-Myc affinity beads (Sigma) as indicated, resolved by SDS-PAGE. Gel bands were isolated, digested with trypsin and the peptides were analyzed by MS on a Micromass Q-T of hybrid quadrupole/time-of-flight mass spectrometer with a Nanoelectrospray source. Raw data files were processed using the MassLynx ProteinLynx software.

### Real-time PCR for gene expression

cDNA templates used for real-time PCR were reverse transcribed from RNA isolated from wild-type C2C12 myoblasts. RNA was purified using HiPure Total RNA Mini Kit (Magen) according to the manufacturer’s recommendations. First strand cDNA was synthesized using TransScript All-in-One First-Strand cDNA Synthesis SuperMix for qPCR Kit (TransGen Biotech) according to the manufacturer’s recommendations. Quantitative real-time PCR was performed using SYBR green reagent (CWBiotech) in the Roche LightCycler 480 machine. Expression values were normalized to *β*-*Actin*. At least three independent experiments were performed for each gene. Data were analyzed using 2^−∆∆*Ct*^. The average values were given as the mean ± SD. The primers were shown below. F-MyoD: 5′-GGCTACGACACCGCCTACTA-3′; R-MyoD: 5′-CTGGGTTCCCTGTTCTGTGT-3′. F-Myf5: 5′-TGAGGGAACAGGTGGAGAAC-3′; R-Myf5: 5’-AGCTGGACACGGAGCTTTTA-3′. F-β-Actin: 5′-ATGGTGGGAATGGGTCAGAAG-3′; R-β-Actin: 5′-CCATGTCGTCCAGTTGGTAA-3′.

### Co-immunoprecipitation (Co-IP) and Western blotting analyses

For Western blotting, C2C12 myoblasts were lysed in RIPA buffer (CWBiotech), and proteins were analyzed by ECL Western blotting detection system (Tanon-5200). For Co-IP assays, cells were lysed in BC300 buffer (10% glycerol, 0.2 mM EDTA, 0.3 mM KCl, protease inhibitor cocktail [CWbiotech], Phenylmethanesulfonyl fluoride [PMSF] [Meilun Biotech], 25 mM Tris-HCl [pH 7.9]) containing 0.1% NP40. The cell lysates were then sonicated, centrifuged at 16,000 g in 4 °C for 10 min. 200 μL supernatants were transferred to a new EP tube used as total protein (Input sample). Remaining supernatants were used to prepare IP samples. Supernatants were incubated with Protein A/G Sepharose (Sigma) in 4 °C for 1 h to remove impurities. The samples were then incubated with Anti-Flag/Anti-c-Myc affinity beads (Sigma) or indicated antibodies in 4 °C overnight for enrichment of target proteins. Following being washed by BC300 buffer, immunoprecipitated proteins were eluted by 1× SDS sample buffer (CWBiotech) and analyzed by ECL Western blotting detection system (Tanon-5200). Immunoprecipitation assays from limb nuclear extracts were done in BC200 buffer (10% glycerol, 0.2 mM EDTA, 0.2 mM KCl, protease inhibitor cocktail [CWbiotech], Phenylmethanesulfonyl fluoride [PMSF] [Meilun Biotech], 25 mM Tris-HCl [pH 7.9]) containing 0.1% NP40. IP Samples were made with Anti-Msx1 antibody followed by precipitation with Protein G beads (Sigma) at 4 °C for 1 h. Following being washed by BC200 buffer, immunoprecipitated proteins were eluted using 1× SDS sample buffer (CWBiotech) and analyzed by immunoblotting using an ECL Western blotting detection system (Tanon-5200). The antibodies used for Co-IP and Western blotting were shown below. Anti-Flag (Mouse mAb, Sigma # F3165, 1:10,000), Anti-c-Myc-Peroxidase antibody (Rabbit pAb, Sigma # A5598, 1:3000), Anti-Msx1 (Mouse mAb, Covance # MMS-261R, 1:1000 [Western blotting], 1:80 [Co-IP]), Anti-β-Actin (Mouse mAb, CWBiotech # CW0096M, 1:4000), Anti-α-tubulin (Mouse mAb, GeneTex # GTX628802, 1:5000), Anti-Emerin (Rabbit pAb, Bioworld Technology # BS6162, 1:1000), Anti-H3K9me2 (Mouse mAb, Abcam # ab1220, 1:1000), Goat Anti-Mouse IgG, HRP Conjugated (CWBiotech # CWO102S, 1:5000), Goat Anti-Rabbit IgG, HRP Conjugated (Abmart # M21002, 1:3000).

### Immunofluorescence (IF) assays

293T cells or C2C12 myoblasts were seeded on 1-well glass bottom plate (NEST). When cell confluence was 40%, IF assays were performed. Cells were fixed in 4% paraformaldehyde (PFA) (Invitrogen) for 20 min at room temperature, then rinsed with 1× phosphate buffer saline (1× PBS) (Gibco) to wash off the PFA and permeabilized by 0.2% Triton X-100 (Sigma) for 20 min at room temperature. After washing off permeabilized solution with 1× PBS, cells were blocked with 10% goat serum (Gibco) in 1× PBS, and incubated with primary antibodies at 37 °C for 2 h followed by incubation with Alexa Fluor 488 and/or Alexa Flour 555 secondary antibodies (Thermo Fisher) at 37 °C for 1 h after washing off non-specific primary antibodies with 1× PBS. Cell nuclear was stained by DAPI (Thermo Fisher) for 3–5 min at room temperature after non-specific secondary antibodies being washed off by 1× PBS. 1× PBS was used to wash off non-specific DAPI, and IF staining was visualized by fluorescence inversion microscope system (Zeiss). The antibodies used in IF assays were shown below. Anti-Flag (Mouse mAb, Sigma # F3165, 1:5000), Anti-DYKDDDK-Tag (Mouse mAb, Abmart # M20008, 1:1000), Anti-Emerin (Rabbit pAb, Bioworld Technology # BS6162, 1:300), Anti-Ezh2 (Rabbit pAb, Millipore # 07-689, 1:250), Anti-H3K27me3 (Rabbit pAb, Millipore # 07-449, 1:500), Anti-MHC (Mouse mAb, Developmental Studies Hybridoma Bank MF20, 1:200), Goat anti-Mouse IgG (H + L) Highly Cross-Adsorbed Secondary Antibody, Alexa Fluor 555 (Thermo Fisher, # A21424, 1:1000), Goat anti-Rabbit IgG (H+L) Cross-Adsorbed Secondary Antibody, Alexa Fluor 488 (Thermo Fisher, # A11008, 1:1000).

The subnuclear localization was quantitated using ImageJ (http://rsb.info.nih.gov/ij/) [37]. A line was drawn from the nuclear periphery to the nuclear center, and along this line, the fluorescence intensity was recorded; the pixel values versus radial position were used to generate the quantitative plot. As indicated cell data were shown for individual representative cells.

### Acquirement of epiCRISPR/Cas9 knockdown cells

The epiCRISPR/Cas9 plasmid with Emerin-gRNA was constructed according to the protocol applied by Xie et al [36]. Two gRNAs targeting Emerin DNA sequence were designed by an online tool (http://crispr.mit.edu/). The epiCRISPR/Cas9 with Emerin-gRNA plasmids were transfected into C2C12 myoblasts when cell confluence was over 70% using Lipofectamine 2000 reagent (Invitrogen) according to the manufacturer’s recommendations. Cells were selected by puromycin (3 μg/mL) (Sangon Biotech) 1 day after transfection. When cell confluence was 50%, puromycin was removed from DMEM supplemented with 10% FBS to let the cells with epiCRISPR/Cas9 plasmids proliferate well. When cell confluence was 90%, they were used for cryopreservation and evaluation of knockdown efficiency by Western blotting.

### Statistical analysis

At least three independent experiments were performed for each assay. The average values of the parallel experiments were given as the mean ± SD. Comparison of differences between two groups was carried out by Student’s *t*-test. Significance was defined as *p* < 0.01 (***p < 0.0001, **p < 0.001, *p < 0.01).

## Results

### Msx1 associates with Emerin via the homeodomain

**Fig. 1:**
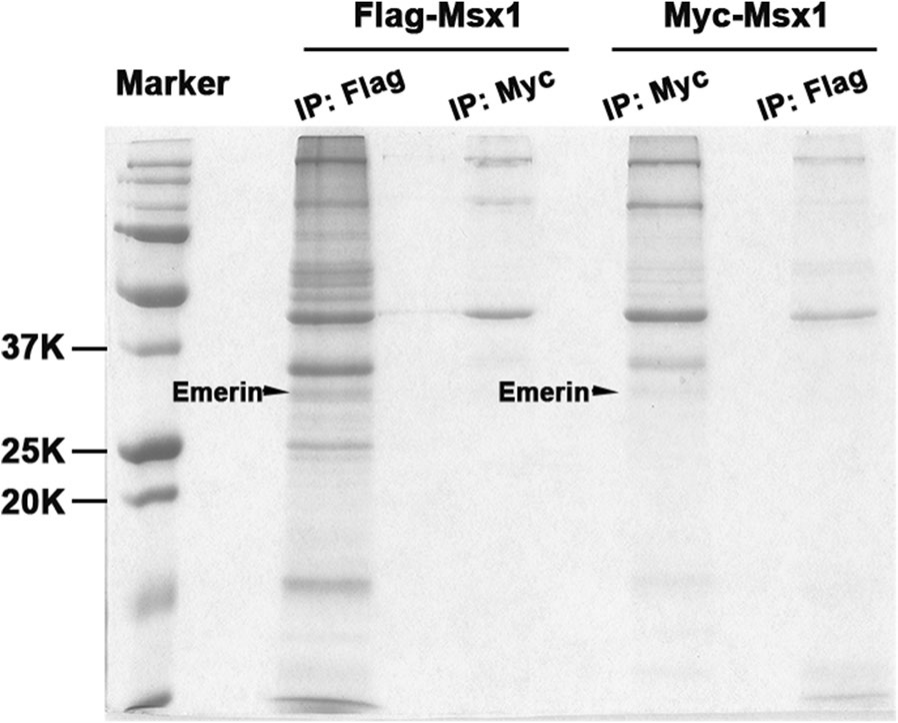
Msx1 associates with Emerin in C2C12 myoblasts. IP-MS using C2C12 myoblasts with exogenous Flag-Msx1 or Myc-Msx1

**Fig. 2:**
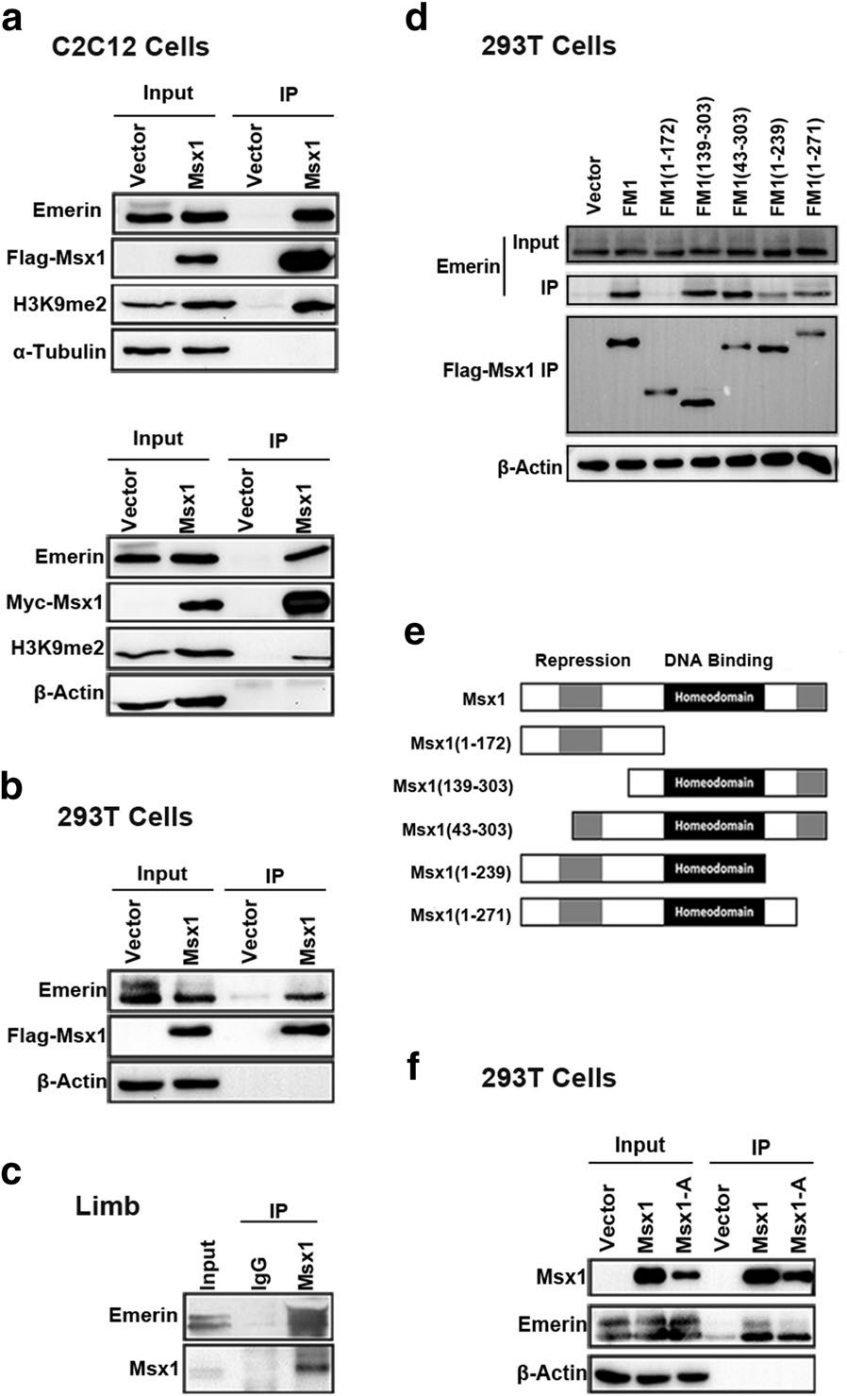
Msx1 associates with Emerin via the homeodomain, regardless of DNA binding ability. **a** C2C12 myoblasts were overexpressed with Flag-Msx1 or Myc-Msx1. Whole cell lysates of C2C12 myoblasts with overexpressed Flag-Msx1 or Myc-Msx1 were immunoprecipitated with anti-Flag beads or anti-c-Myc beads respectively followed by immunoblotting for Emerin or other proteins as indicated. H3K9me2 was a positive control. **b** 239T cells overexpressed with exogenous Flag-Msx1 were used for Co-IP. Whole cell lysates of 293T cells with overexpressed Flag-Msx1 were immunoprecipitated with anti-Flag beads followed by immunoblotting for Emerin, Flag and β-Actin. **c** Lysates of embryonic forelimb (E11.5) were immunoprecipitated with anti-Msx1 antibody followed by immunoblotting for Emerin and Msx1. **d** Domain mapping analyses. 293T cells overexpressed with Msx1 protein fragments were used for immunoprecipitation followed by immunoblotting for Emerin, Flag and β-Actin. **e** Schematic representation of Msx1 protein fragments according to its functional domain. **f** 293T cells were overexpressed with exogenous Msx1 or Msx1-A (K174A, R176A and F179A). Whole cell lysates were immunoprecipitated with Anti-Msx1 antibody followed by immunoblotting for Emerin, Msx1 and β-Actin

Our results of IP-MS using nuclear extracts from C2C12 myoblasts overexpressing exogenous Msx1 fused with tags showed that Msx1 associated with Emerin (Fig. 1). To confirm the possible interaction between Msx1 and Emerin, we firstly overexpressed Msx1 fused with tags in C2C12 myoblasts and 293T cells to perform Co-IP respectively (Fig. 2a, b). Our data showed that Msx1 associated with Emerin both in C2C12 myoblasts and 293T cells, regardless of applying Anti-Flag beads or Anti-c-Myc beads to enrich the Msx1-interacted proteins (Fig. 2a, b). Furthermore, results of Co-IP using Anti-Msx1 antibody to enrich Msx1-interacted proteins in mouse developing limbs (E11.5) identified the interaction between these two proteins in vivo (Fig. 2c), indicating the potential biological significance of these two proteins’ association.

Msx1 is consisted of multiple functional domains, which contribute to DNA binding ability, transcriptional repression function and subnuclear localization of Msx1 [33–35]. We obtained five Msx1 protein fragments according to its functional domains based on previous works [33–35]. Utilizing these protein fragments, we identified that the homeodomain of Msx1 was essential for the interaction between itself and Emerin (Fig. 2d, e). Considering that the homeodomain endows Msx1 with DNA binding ability [35], we wondered whether the interaction between Msx1 and Emerin relied on DNA. We used Msx1A (there are three mutations in amino acids’ sequence of Msx1, which are K174A, R176A, and F179A), a Msx1 mutant losing DNA binding ability, to investigate whether the interaction was mediated by DNA. Co-IP data in 293T cells showed that DNA bound by Msx1 was dispensable for the interaction between Msx1 and Emerin (Fig. 2f). So we believed that the homeodomain was essential for the interaction between Msx1 and Emerin, regardless of Msx1’s DNA binding ability.

**Fig. 3:**
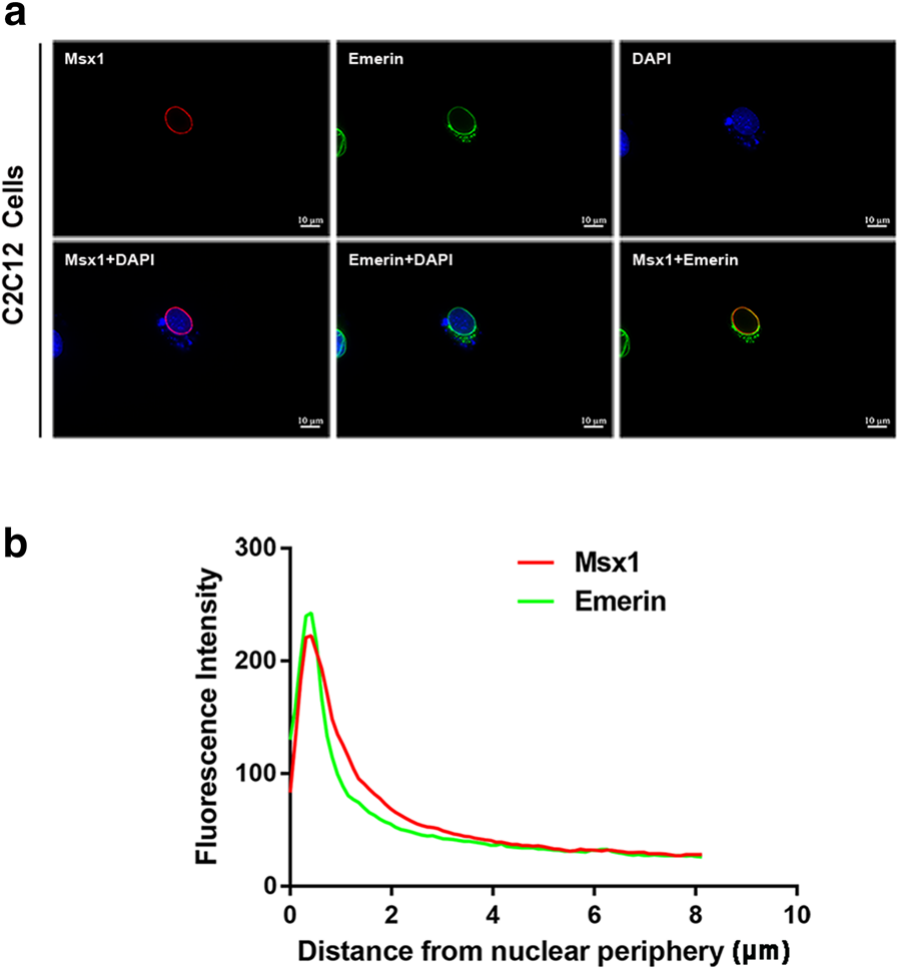
Msx1 and Emerin are co-localized at the nuclear periphery. **a** C2C12 myoblasts expressing exogenous Flag-Msx1 were used for IF assays. **b** Quantitative analysis with ImageJ showed representative data from three independent assays. Scale bars represented 10 µm

Since Msx1 tends to be enriched at the nuclear periphery in myogenic lineage [33–35], we next validated the co-localization of Msx1 and Emerin in C2C12 myoblasts. Our immunofluorescence (IF) data showed that exogenous Msx1 and Emerin had the same spatial localization in C2C12 myoblasts, both of which were localized at the nuclear periphery in cells overexpressed with exogenous Flag-Msx1 (Fig. 3a, b). These results suggested their interaction most likely happened on the nuclear membrane, which is consistent with Emerin’s nuclear localization in myogenic lineage as an inner nuclear membrane protein [11], and our previous data that Msx1 is located at the nuclear periphery both in C2C12 myoblasts and mouse limb buds [33–35].

Taken together, we showed that Msx1 interacted with Emerin at the nuclear periphery, which required the homeodomain of Msx1, but the interaction may not be mediated by DNA. Importantly, the interaction also existed in mouse developing limbs, suggesting the association between Msx1 and Emerin was required for in vivo functionality, like the maintenance of undifferentiated state of myogenic precursors in developing limb buds. Indeed, studies have shown that *Msx* genes are essential for the proliferation of undifferentiated cells in limb buds [38].

### Emerin is indispensable for the nuclear periphery localization of Msx1

**Fig. 4:**
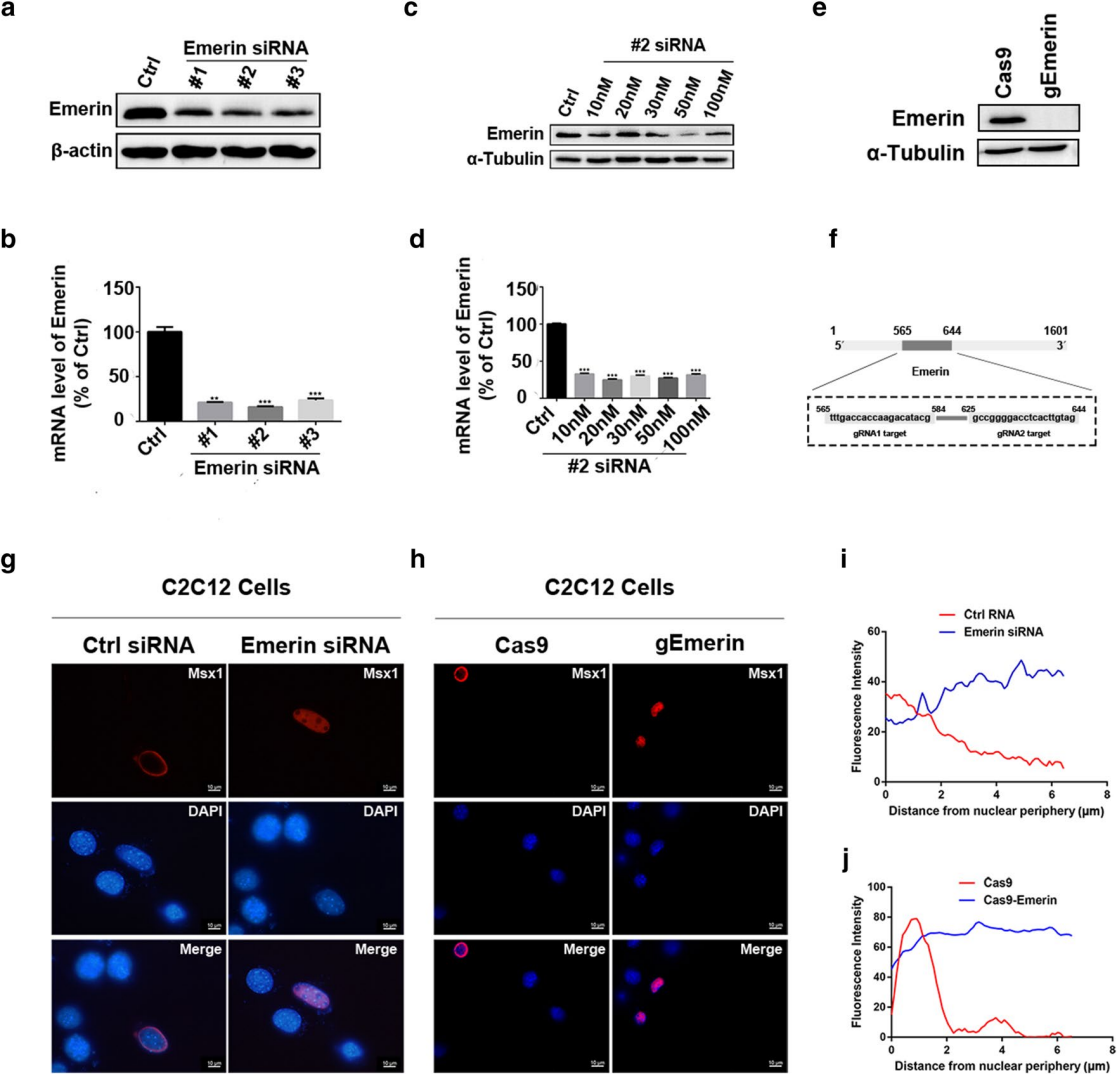
Emerin contributes to the nuclear periphery localization of Msx1. **a**–**d** Knockdown Emerin in C2C12 myoblasts using siRNAs. **a** The expression levels of Emerin after using different siRNAs to knockdown Emerin in C2C12 myoblasts. **b** mRNA levels of Emerin after using different siRNAs to knockdown Emerin in C2C12 myoblasts. Data are shown as the relative expression level in cells with the Emerin siRNA or the control. Values are the mean ± SD. ***p < 0.0001, **p < 0.001, *p < 0.01. **c** The expression levels of Emerin after using Emerin#2 siRNA in different transfection concentrations to knockdown Emerin in C2C12 myoblasts. **d** mRNA expression levels of Emerin after using Emerin#2 siRNA in different transfection concentrations to knockdown Emerin in C2C12 myoblasts. Data are shown as the relative expression level in cells with the Emerin siRNA or the control. Values are the mean ± SD. ***p < 0.0001, **p < 0.001, *p < 0.01. **e**, **f** Knockdown Emerin in C2C12 myoblasts using epiCRISPR/Cas9 system. **e** The expression levels of Emerin using epiCRISPR/Cas9 system to knockdown Emerin in C2C12 myoblasts. **f** Schematic diagram of two guide RNAs. **g**–**j** IF data showing the subnuclear localization of Msx1 altered from the nuclear periphery to interior nuclear after Emerin was knockdown in C2C12 myoblasts. **g** IF assays performed in C2C12 myoblasts transfected with Emerin#2 siRNA. **h** IF assays performed in C2C12 myoblasts transfected with epiCRISPR/Cas9 plasmids. **i**–**j** Quantification of Msx1 localization from G and H was done using ImageJ. Scale bars represented 10 µm

Studies have shown that Msx1 is localized at the nuclear periphery in myogenic lineage in vitro and in vivo, and its subnuclear localization is relevant to target genes’ expression states [30, 33–35]. Since Emerin is localized at the nuclear periphery itself, and we confirmed the interaction between Msx1 and Emerin, we speculated that Emerin contributed to Msx1’s precise subnuclear localization. To test this idea, we utilized siRNA and epiCRISPR/Cas9 system respectively to knockdown or delete Emerin in C2C12 myoblasts [36] (Fig. 4a–f), and investigated the subnuclear localization of Msx1 in Emerin deficient cells (Fig. 4g–j).

For siRNA silencing, we observed that Emerin#2 siRNA efficiently silenced the transcription of Emerin in C2C12 myoblasts among three Emerin siRNAs (Fig. 4a, b). The expression level of Emerin in C2C12 myoblasts transfected with Emerin#2 siRNA was intensively reduced (> 80%, *p* < 0.0001, 10 nM siRNA were used for screening) (Fig. 4b). Then we identified that the most effective working concentration for Emerin#2 siRNA was 50 nM (Fig. 4c, d). The signal of Emerin protein was obviously weakened when 50 nM Emerin#2 siRNA was transfected to C2C12 myoblasts (Fig. 4c), and the transcription of Emerin was inhibited over 70% (*p* < 0.0001) (Fig. 4d). Given that siRNA was not capable of eliminating target genes’ expression in a single cell, we implemented the knock-out of Emerin on a single cell level by transfecting epiCRISPR/Cas9 system to C2C12 myoblasts [36]. Here we emphasized that though epiCRISPR/Cas9 system was able to delete our target gene on a single cell level, it was technically tough to ensure the knock-out efficiency in all cells of a cell population. Since epiCRISPR/Cas9 system was applied to cell populations in our work, we still described it as knockdown of Emerin in this writing (Fig. 4e, f). For epiCRISPR/Cas9 system, we designed two guide RNAs (gRNAs) for the DNA sequence encoding Emerin to ensure the knockdown efficiency of Emerin by editing both of gRNAs at the same time, and there were 40 base pairs in mice genome between these two gRNAs’ target sites (Fig. 4f). Barely no signal of Emerin was detected in epiCRISPR/Cas9 knockdown group showed by our Western blotting data, suggesting that Emerin was efficiently knockdown in epiCRISPR/Cas9-transfected C2C12 myoblasts (Fig. 4e).

Then we performed IF in C2C12 myoblasts transfected with Emerin#2 siRNA and epiCRISPR/Cas9 system respectively. As what we had expected, the distribution of exogenous Msx1 altered from the nuclear periphery to interior nuclear when Emerin was repressed or completely inhibited (Fig. 4g–j). For siRNA group, more than 100 cells transfected with control siRNA or Emerin#2 siRNA were analyzed respectively. We observed that Msx1 was localized at the nuclear periphery in over 90% of cells with control siRNA, while evenly distributed in interior nuclear in 100% of cells with Emerin#2 siRNA. For epiCRISPR/Cas9 group, 100 cells transfected with Cas9 plasmids or gEmerin plasmids were analyzed respectively. Msx1 was localized at the nuclear periphery in about 80% control cells but only 20% Emerin knockdown cells. These observations suggested that a certain amount of Emerin was required for the nuclear periphery localization of Msx1 in a single C2C12 myoblast. Each time before we performed IF using Emerin knockdown cells, the expression level of Emerin were checked through Western blotting to ensure the maintenance of knockdown efficiency (data not shown).

Consistently, our previous data have shown that the Msx1 protein fragment (1–172) is distributed in the interior nuclear [33–35]. And in this work, we identified that the Msx1 protein fragment (1–172) didn’t interact with Emerin. Combined these two results with our IF data using C2C12 myoblasts lacking Emerin, we concluded that Msx1’s precise subnuclear localization in myogenic lineage required Emerin, and their association was necessary for the nuclear periphery localization of Msx1.

### Emerin impairs the redistribution of H3K27me3 mediated by Msx1 in C2C12 myoblasts

**Fig. 5:**
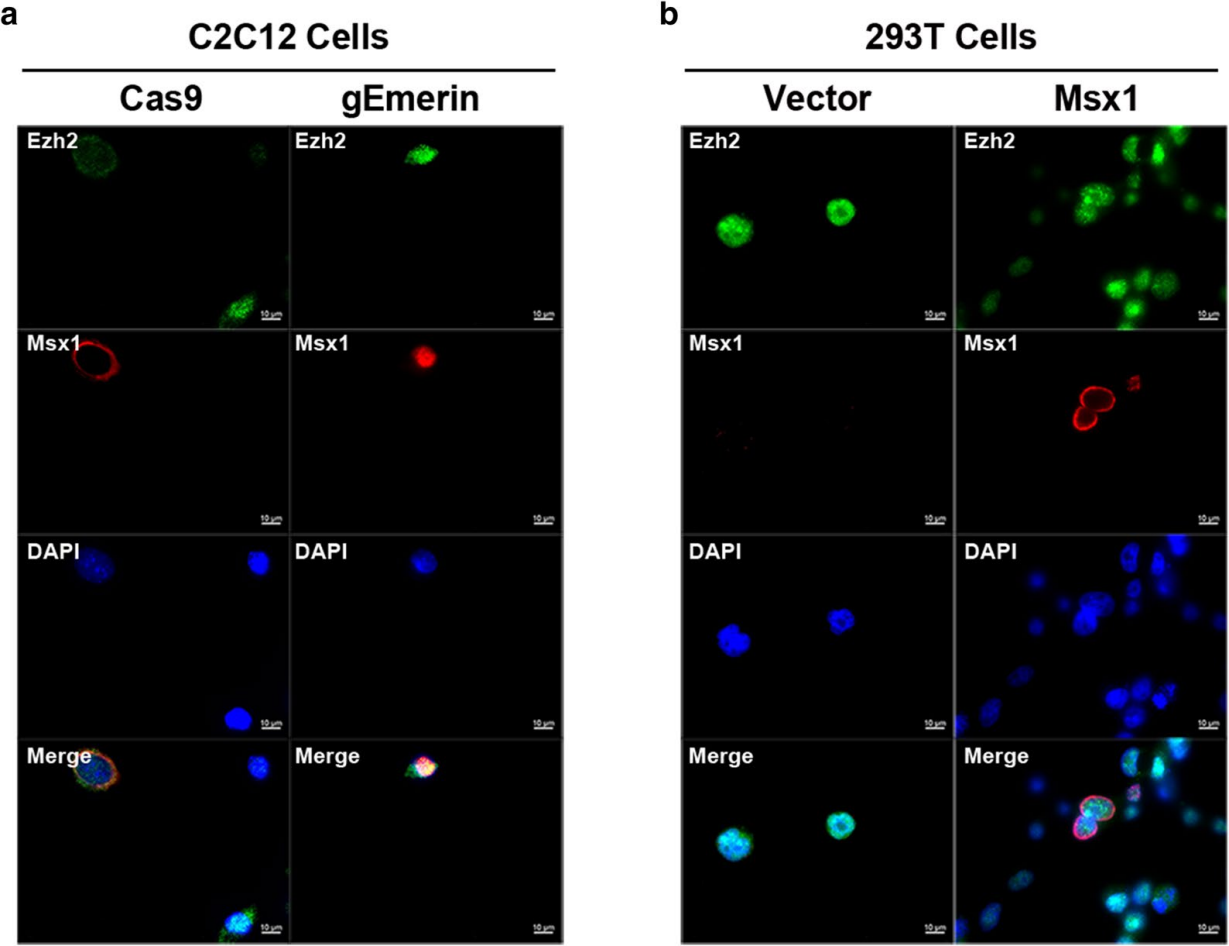
The subnuclear localization of Ezh2 alters from the nuclear periphery to interior nuclear after Emerin is knockdown in C2C12 myoblasts. **a** IF assays using C2C12 myoblasts with exogenous Flag-Msx1. Emerin was knockdown in C2C12 myoblasts using epiCRISPR/Cas9 system. Ezh2 was no longer localized at the nuclear periphery after Emerin was knockdown in C2C12 myoblasts. **b** IF assays using 293T cells with exogenous Flag-Msx1. Exogenous Msx1 was localized at the nuclear periphery in 293T cells, but Ezh2 distributed at the interior nuclear. Scale bars represented 10 µm

**Fig. 6:**
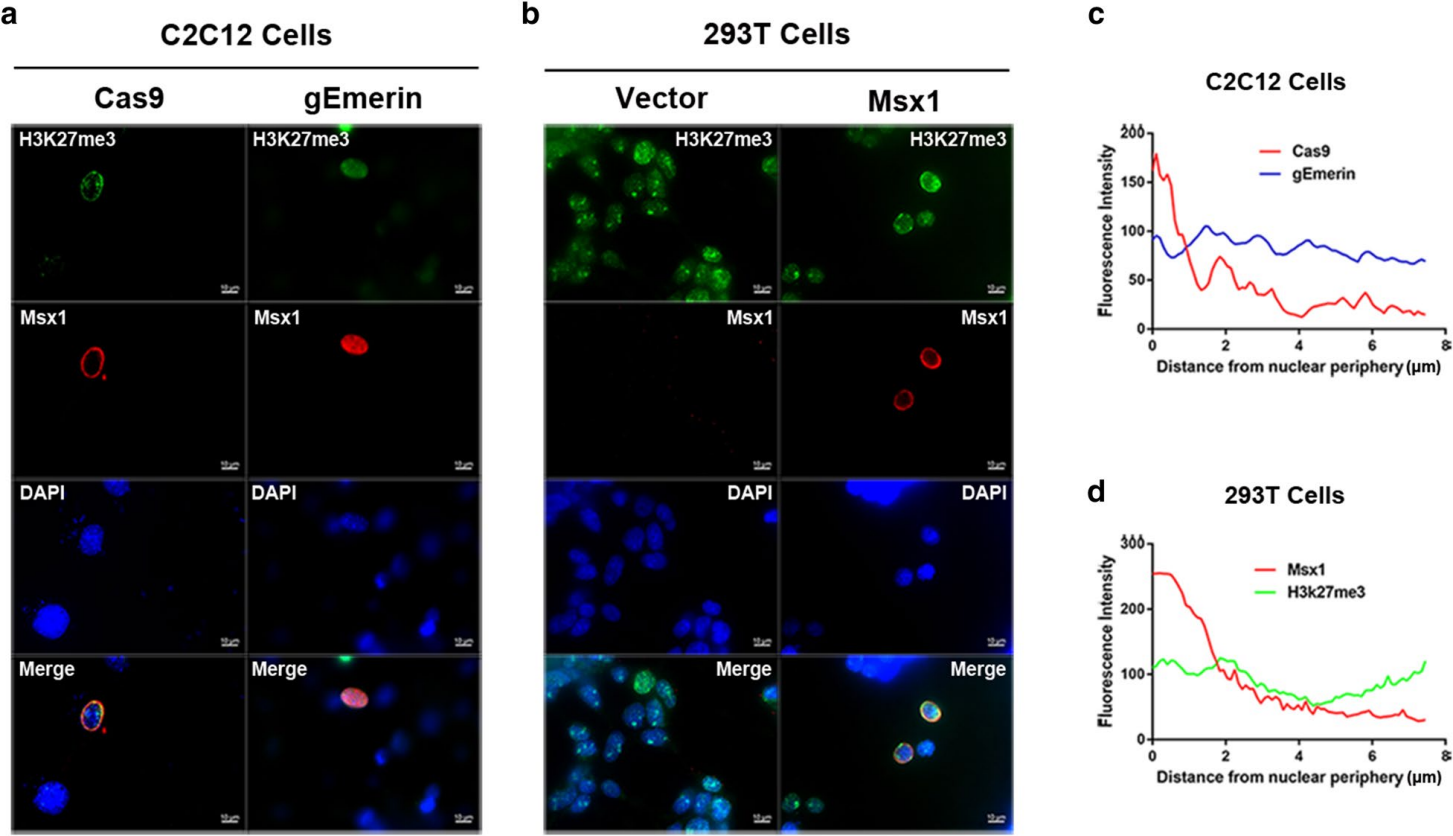
The subnuclear localization of H3K27me3 alters from the nuclear periphery to interior nuclear after Emerin is knockdown in C2C12 myoblasts. **a** IF assays using C2C12 myoblasts with exogenous Flag-Msx1. Emerin was knockdown in C2C12 myoblasts using epiCRISPR/Cas9 system. H3K27me3 was no longer localized at the nuclear periphery after Emerin was knockdown in C2C12 myoblasts. **b** IF using 293T cells with exogenous Flag-Msx1. H3K27me3 distributed at the interior nuclear in 293T cells. **c** Quantification of H3K27me3 localization in C2C12 myoblasts was done using ImageJ. **d** Quantification of H3K27me3 localization in 293T cells was done using ImageJ. Scale bars represented 10 µm

Our previous studies have shown that Msx1 recruits histone methyltransferase Ezh2 to target repressive genes and redistributes histone mark H3K27me3, which is modified by Ezh2, from interior nuclear to the nuclear periphery during inhibition of myogenesis [35]. Since the subnuclear localization of Msx1 relied on its interaction with Emerin, and Msx1-induced nuclear periphery-enrichment of Ezh2 and repressive mark H3K27me3 in C2C12 myoblasts, we speculated that histone methyltransferase Ezh2 and histone mark H3K27me3 would be no longer localized at the nuclear periphery in Emerin deficient C2C12 myoblasts overexpressed with exogenous Msx1. Indeed, we observed that when Emerin was deleted in a single C2C12 myoblast by epiCRISPR/Cas9 system, the histone methyltransferase Ezh2 moved from the nuclear periphery to interior nuclear (Fig. 5a), so did the repressive histone mark H3K27me3 (Fig. 6a, c). For the subnuclear localization of Ezh2 or H3K27me3, 100 control cells and 100 Emerin knockdown cells were analyzed respectively. Ezh2 and H3K27me3 were localized at the nuclear periphery in more than 80 control cells but less than 20 Emerin knockdown cells.

Besides, in the majority of 293T cells (over 90% in 100 cells), we observed that though exogenous Msx1 was localized at the nuclear periphery as in C2C12 myoblasts, Ezh2 was not co-localized with Msx1 (Fig. 5b). Consistently, the repressive mark H3K27me3 was evenly distributed in the inner nuclear in 293T cells (Fig. 6b, d). These results indicated that the redistribution of histone methyltransferase Ezh2 and histone mark H3K27me3 induced by Msx1 was cell lineage specific, and the redistribution of repressive methylated marks mediated by Msx1 was dependent on cell context. Actually, not only did the distribution of Msx1’s protein partners rely on cell type, Msx1’s distribution was different from cell type to cell type [35]. These reminded us of distinct ways Msx1 applied to regulate target genes in individual cell types.

Thus we concluded that the deficiency of Emerin destroyed the well-organized distribution of Msx1 and its protein partners in C2C12 myoblasts, and the redistribution of histone methyltransferases and their repressive histone marks mediated by Msx1 was cell lineage specific.

### Emerin is required for Msx1 to inhibit myogenesis

Next we investigated whether the interaction between Msx1 and Emerin assisted Msx1 with target myogenic regulatory genes’ repression and inhibition of myogenesis. We speculated that Emerin participated in histone methyltransferases recruitment and repressive histone marks redistribution mediated by Msx1 to inhibit myogenic differentiation.

**Fig. 7:**
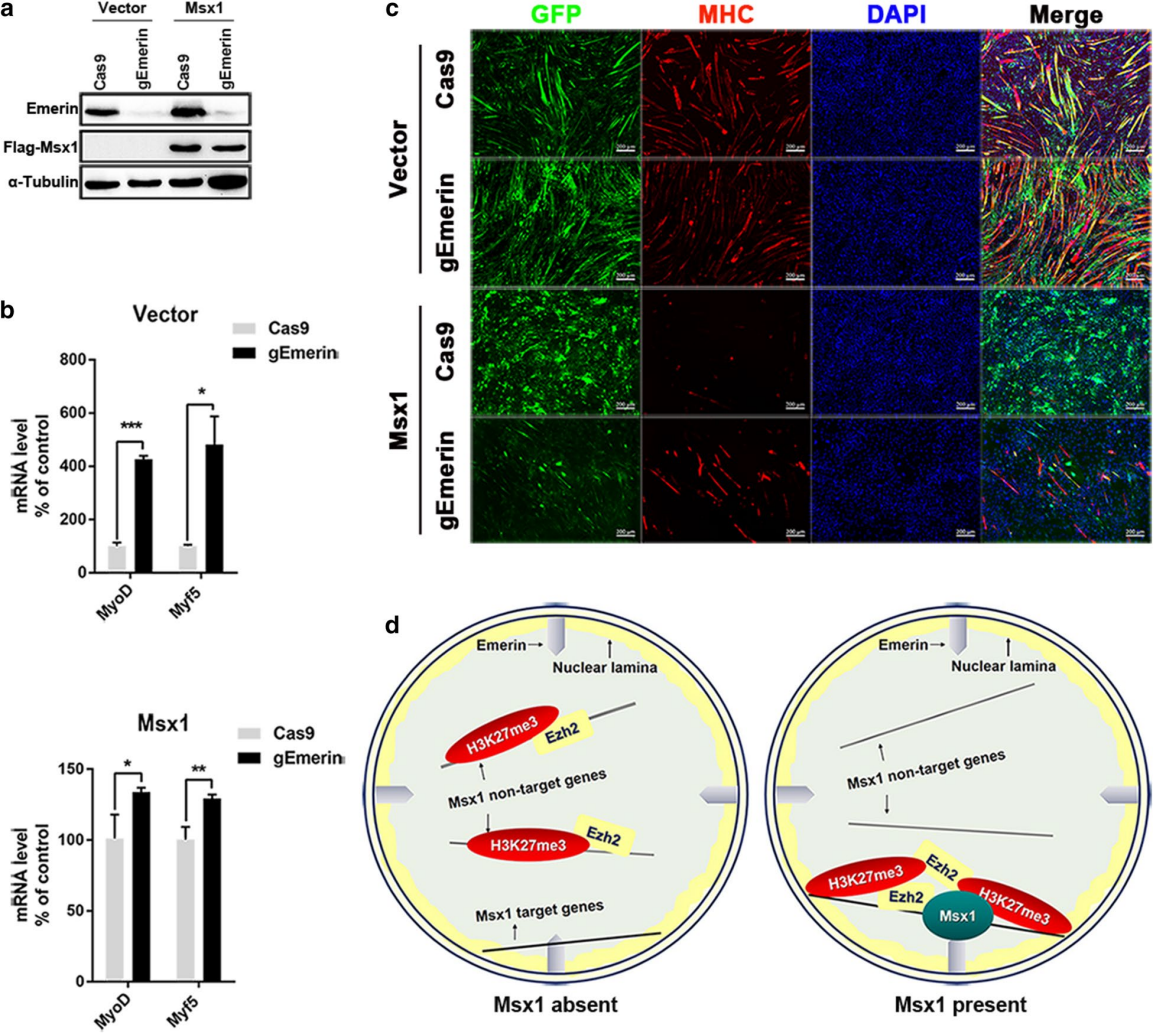
The association between Msx1 and Emerin is necessary for inhibition of myoblasts’ differentiation and the repression of myogenic targets. **a** Western blotting assays. To ensure Flag-Msx1 was successfully overexpressed and Emerin was efficiently knockdown in C2C12 myoblasts used in real-time PCR (**b**) and IF assays (**c**). **(b)** mRNA levels of Msx1 target myogenic genes *MyoD* and *Myf5*. Data were shown as the relative expression levels in cells overexpressing Msx1 or the control vectors. Values were the mean ± SD. ***p < 0.0001, **p < 0.001, *p < 0.01. **c** IF assays. C2C12 myoblasts were transfected with epiCRISPR/Cas9 plasmids firstly to acquire Emerin deficient cells (transfected with Cas9-gEmerin) and control cells (transfected with Cas9). Then these cells were infected by retrovirus with plasmids expressing Flag-Msx1 and GFP (vectors overexpressing Msx1) or only GFP (control vectors). IF assays were performed after induction by DMEM with 2% horse serum for 3–5 days as detected using antibody for myosin heavy chain (MHC) and DAPI. Cells expressing GFP were successfully infected by retrovirus. Scale bars represented 200 µm. **d** Schematic diagram of Ship-to-Anchor model. Emerin associated with Msx1 at the nuclear periphery to help Msx1 with the recruitment of histone methyltransferase Ezh2 to target genes

To identify our hypothesis, we investigated the alteration of myogenic differentiation states and target genes’ expression levels in C2C12 myoblasts with Emerin knockdown and control cells. We utilized epiCRISPR/Cas9 system to knockdown Emerin in C2C12 myoblasts firstly, then overexpressed Msx1 in these cells to obtain cells which were deficient in the expression of Emerin while overexpressed Msx1 (Fig. 7a). When Emerin was knockdown in C2C12 myoblasts, myogenic regulatory genes *MyoD* and *Myf5* were up-regulated in varying degrees, about threefold increase (*p* < 0.0001) and fourfold increase (*p* < 0.01) respectively (Fig. 7b), which was consistent with Emerin’s function in repressing *MyoD* and *Myf5* through forming a complex with Lmo7 to reduce inner nuclear Lmo7, which is an activator of *MyoD* and *Myf5* [39, 40]. In Msx1-overexpressed C2C12 myoblasts, the deficiency of Emerin attenuated the repression of *MyoD* (about 0.3-fold increase, *p* < 0.01) and *Myf5* (about 0.3-fold increase, *p* < 0.001) mediated by Msx1, and led to a partial differentiation (Fig. 7b, c). Here we only observed a partial but not complete differentiation of C2C12 myoblasts. This was possibly attributed to the existence of inner nuclear membrane proteins besides Emerin, which may also contribute to Msx1’s nuclear periphery localization. Indeed, in our IP-MS data using C2C12 myoblasts overexpressed with Msx1, some other inner nuclear membrane proteins also have been identified (data not shown).

Based on these results, we proposed a Ship-to-Anchor model to illustrate the role of Emerin in inhibition of myogenesis mediated by Msx1 (Fig. 7d). In this model, Emerin acted as an anchor at the nuclear periphery to maintain the precise subnuclear localization of Msx1. At the time Msx1 was highly expressed in C2C12 myoblasts, Msx1 was locked by Emerin at the nuclear periphery, then recruited histone methyltransferase Ezh2 to target myogenic regulatory genes to ensure the repression of target genes through maintaining chromosomes in a repressive state.

## Discussion

It’s intriguing that Msx1 tends to be localized at the nuclear periphery in myogenic lineage in vitro and in vivo, in which Msx1’s repressive genes are localized at the nuclear periphery as well. Our laboratory has shown that Msx1 is able to recruit histone methyltransferases to target myogenic regulatory genes to inhibit their expression through altering the modification states of chromosomes [33–35]. However, the mechanisms underlying Msx1 and its protein partners’ nuclear periphery localization are unclear.

In this work, we report an inner nuclear membrane protein, Emerin, acting as an anchor at the inner nuclear membrane to guarantee the precise nuclear position of Msx1 and its protein partners. A Ship-to-Anchor model is proposed according to our data. We consider Emerin as an anchor to keep Msx1 and its protein partners at the nuclear periphery stably in order to maintain the cells expressing high level of Msx1 in an undifferentiated state (Fig. 7d). The interaction between Msx1 and Emerin is substantially validated through Co-IP both in vitro and in vivo in our studies, and IF data indicate their interaction occurs at the nuclear periphery. The deficiency of Emerin impairs inhibition of myogenic differentiation mediated by Msx1, since Emerin is essential for Msx1 in the repression of target myogenic regulatory genes through assisting Msx1 with the recruitment of histone methyltransferases to these genes at the nuclear periphery specifically in C2C12 myoblasts.

In our studies, the protein complexes consisting of Msx1, Ezh2, and H3K27me3 enrich at the nuclear periphery specifically in C2C12 myoblasts with exogenous Msx1, but not in 293T cells, suggesting the mechanism we revealed is cell context dependent. The explanation to this phenotype is possibly that Msx1 cooperates with specific protein partners to regulate genes’ expression in different cell types. Accordingly, our previous studies have shown that even the localization of Msx1 is inhomogeneous in different cell lineages. Like in neural tube, Msx1 is evenly distributed in interior nuclear [35]. These remind us that diverse target genes and distinct biological contexts in different cell lineages determine the way a transcriptional factor adopts to regulate the expression of target genes.

For Emerin, studies from Demmerle and collaborators have shown its capability of regulating genes’ position under the help of other factors in myogenesis [27]. They performed chromatin immunoprecipitation (ChIP) to verify that myogenic regulatory loci *Myf5* and *MyoD* are associated with Emerin in proliferating C2C12 myoblasts [27]. Consistently, our previous studies have shown that myogenic regulatory genes, such as *Myf5*, *MyoD*, are localized at the nuclear lamina in C2C12 myoblasts [33–35]. But in this work, we emphasize the role of Emerin after exogenous Msx1 is introduced to C2C12 myoblasts. Our present studies highlight that the stable maintenance of undifferentiated state mediated by Msx1 in C2C12 myoblasts thanks to the association between Msx1 and Emerin, since the enrichment of repressive histone methylated marks occurs after overexpressing exogenous Msx1, the deficiency of Emerin directly disturbs Msx1 and its protein partners’ nuclear periphery localization.

Though overexpressing Msx1 in C2C12 myoblasts is an in vitro model, some evidence might indicate the in vivo significance of our studies. For instance, in the limbs of E10.5 mouse embryos, Msx1 is enriched at the nuclear periphery in the proliferating limb bud cells [35], which is paralleled with the phenotypes we observed in C2C12 myoblasts with exogenous Msx1. Besides, Msx1 is re-expressed in the proliferating muscle satellite cells during skeletal muscle regeneration, and locates at the nuclear periphery in these activated cells as well [30]. Furthermore, it’s well-characterized that Msx1 prevents the pre-maturation of myogenic progenitor cells in skeletal muscle development, and Msx1’s expression level is reduced as the myogenic differentiation starts [41, 42]. Combining these in vivo data with the facts that overexpression of Msx1 transverses myotube to pluripotent mononucleated cells [43, 44], and Msx1’s overexpression inhibits terminal differentiation of C2C12 myoblasts and transforms C2C12 myoblasts into a myogenic precursor-like state [31, 43, 44], we consider that the nuclear periphery localization of Msx1 is relevant to its function in myogenic lineage restriction.

Recent work from Poleshko and collaborators showed that Hdac3 regulates cardiac progenitor cell lineage restriction through interacting with Lap2β, which mediates the association between Hdac3 and nuclear lamina, to control the accessibility of genes related to cardiac progenitors’ differentiation [8]. Hdac3 was defined as a DNA tether in their work, which enlightens us that whether Msx1 performs as a DNA tether in myogenic lineage, similar to the role of Hdac3 in cardiac progenitors. We postulate that during the processes of skeletal muscle development and regeneration, highly expressed Msx1 keeps the chromosomes of myogenic regulatory genes in a condensed state at the nuclear periphery via associating with the inner nuclear membrane protein Emerin in myogenic precursors or activated muscle stem cells. When it comes into a differentiation stage, the decreased expression level of Msx1 releases these target myogenic regulatory genes from the nuclear periphery to interior nuclear, letting myogenic differentiation go on smoothly. Certainly, other nuclear lamina proteins may also contribute to Msx1 and its protein partners’ nuclear periphery localization, which should be further investigated.

Besides, we notice that the nuclear size of C2C12 myoblasts varies in our IF data (Figs. 4, 5, 6). We attribute the heterogeneity of nuclear size to the long-term use of Puromycin to enrich positive cells when using the epiCRISPR/Cas9 system [36]. Actually, C2C12 myoblast are sensitive to Puromycin, which might affect the cell states of C2C12 cells, causing the heterogeneity of nuclear size. However, since the control cells were also treated with the same amount of Puromycin for the same amount of time, the influence of Puromycin could be ignored when we identified the phenotypes. Furthermore, we also observed that the nuclear size of C2C12 myoblasts with Emerin knockdown tended to be smaller than the nuclear size of control cells (Figs. 4, 5, 6). Since Emerin is involved in the stability of nuclear shape [45], we consider this phenotype as the reflection of nuclear shape instability caused by the knockdown of Emerin.

Finally, our studies revealed a candidate nuclear lamina associated protein Emerin, which cooperates with Msx1 to inhibit myogenesis. This provided a mechanistic insight into which Msx1 maintains its protein partners on target genes at the nuclear periphery using C2C12 in vitro models. And we theorized in vivo significance of the nuclear periphery localization of Msx1 and its protein partners, and the association between Msx1 and Emerin. It’s necessary to provide enough in vivo evidence to solidify our model. We hope that our speculation would be validated through applying mouse models to collect direct in vivo evidences in the future.

## References

[1] Towbin BD, Meister P, Gasser SM (2009). The nuclear envelope—a scaffold for silencing?. Curr Opin Genet Dev.

[2] Pickersgill H, Kalverda B, de Wit E, Talhout W, Fornerod M, van Steensel B (2006). Characterization of the *Drosophila melanogaster* genome at the nuclear lamina. Nat Genet.

[3] Reddy KL, Zullo JM, Bertolino E, Singh H (2008). Transcriptional repression mediated by repositioning of genes to the nuclear lamina. Nature.

[4] Shaklai S, Amariglio N, Rechavi G, Simon AJ (2007). Gene silencing at the nuclear periphery. FEBS J..

[5] Engreitz JM, Pandya-Jones A, McDonel P, Shishkin A, Sirokman K, Surka C (2013). The Xist lncRNA exploits three-dimensional genome architecture to spread across the X chromosome. Science.

[6] Wu S, Hu YC, Liu H, Shi Y (2009). Loss of YY1 impacts the heterochromatic state and meiotic double-strand breaks during mouse spermatogenesis. Mol Cell Biol.

[7] Barrales RR, Forn M, Georgescu PR, Sarkadi Z, Braun S (2016). Control of heterochromatin localization and silencing by the nuclear membrane protein Lem2. Genes Dev.

[8] Poleshko A, Shah PP, Gupta M, Babu A, Morley MP, Manderfield LJ (2017). Genome-nuclear lamina interactions regulate cardiac stem cell lineage restriction. Cell..

[9] Helbling-Leclerc A, Bonne G, Schwartz K (2002). Emery-Dreifuss muscular dystrophy. Eur J Hum Genet..

[10] Mendez-Lopez I, Worman HJ (2012). Inner nuclear membrane proteins: impact on human disease. Chromosoma.

[11] Bione S, Maestrini E, Rivella S, Mancini M, Regis S, Romeo G (1994). Identification of a novel X-linked gene responsible for Emery-Dreifuss muscular dystrophy. Nat Genet.

[12] Nagano A, Koga R, Ogawa M, Kurano Y, Kawada J, Okada R (1996). Emerin deficiency at the nuclear membrane in patients with Emery-Dreifuss muscular dystrophy. Nat Genet.

[13] Manilal S, Nguyen TM, Sewry CA, Morris GE (1996). The Emery-Dreifuss muscular dystrophy protein, Emerin, is a nuclear membrane protein. Hum Mol Genet.

[14] Brachner A, Foisner R (2011). Evolvement of LEM proteins as chromatin tethers at the nuclear periphery. Biochem Soc Trans.

[15] Cai M, Huang Y, Ghirlando R, Wilson KL, Craigie R, Clore GM (2001). Solution structure of the constant region of nuclear envelope protein LAP2 reveals two LEM-domain structures: one binds BAF and the other binds DNA. EMBO J..

[16] Lin F, Blake DL, Callebaut I, Skerjanc IS, Holmer L, McBurney MW (2000). MAN1, an inner nuclear membrane protein that shares the LEM domain with lamina-associated polypeptide 2 and emerin. J Biol Chem..

[17] Segura-Totten M, Wilson KL (2004). BAF: roles in chromatin, nuclear structure and retrovirus integration. Trends Cell Biol.

[18] Margalit A, Segura-Totten M, Gruenbaum Y, Wilson KL (2005). Barrier-to-autointegration factor is required to segregate and enclose chromosomes within the nuclear envelope and assemble the nuclear lamina. Proc Natl Acad Sci USA.

[19] Samson C, Petitalot A, Celli F, Herrada I, Ropars V, Le Du MH (2018). Structural analysis of the ternary complex between lamin A/C, BAF and emerin identifies an interface disrupted in autosomal recessive progeroid diseases. Nucleic Acids Res.

[20] Segura-Totten M, Kowalski AK, Craigie R, Wilson KL (2002). Barrier-to-autointegration factor: major roles in chromatin decondensation and nuclear assembly. J Cell Biol..

[21] Haraguchi T, Koujin T, Segura-Totten M, Lee KK, Matsuoka Y, Yoneda Y (2001). BAF is required for Emerin assembly into the reforming nuclear envelope. J Cell Sci.

[22] Wang X, Xu S, Rivolta C, Li LY, Peng GH, Swain PK (2002). Barrier to auto integration factor interacts with the cone-rod homeobox and represses its transactivation function. J Biol Chem..

[23] Holaska JM, Lee KK, Kowalski AK, Wilson KL (2003). Transcriptional repressor germ cell-less (GCL) and barrier to auto integration factor (BAF) compete for binding to Emerin in vitro. J Biol Chem..

[24] Yorifuji H, Tadano Y, Tsuchiya Y, Ogawa M, Goto K, Umetani A (1997). Emerin, deficiency of which causes Emery-Dreifuss muscular dystrophy, is localized at the inner nuclear membrane. Neurogenetics..

[25] Markiewicz E, Venables R, Mauricio Alvarez R, Quinlan R, Dorobek M, Hausmanowa-Petrucewicz I (2002). Increased solubility of lamins and redistribution of Lamin C in X-linked Emery-Dreifuss muscular dystrophy fibroblasts. J Struct Biol.

[26] Fidzianska A, Hausmanowa-Petrusewicz I (2003). Architectural abnormalities in muscle nuclei. Ultrastructural differences between X-linked and autosomal dominant forms of EDMD. J Neurol Sci..

[27] Demmerle J, Koch AJ, Holaska JM (2013). Emerin and histone deacetylase 3 (HDAC3) cooperatively regulate expression and nuclear positions of MyoD, Myf5, and Pax7 genes during myogenesis. Chromosome Res..

[28] Bendall AJ, Abate-Shen C (2000). Roles for Msx and Dlx homeoproteins in vertebrate development. Gene.

[29] Bendall AJ, Ding J, Hu G, Shen MM, Abate-Shen C (1999). Msx1 antagonizes the myogenic activity of Pax3 in migrating limb muscle precursors. Development..

[30] Vojnits K, Pan H, Mu X, Li Y (2015). Characterization of an injury induced population of muscle-derived stem cell-like cells. Sci Rep..

[31] Hu G, Lee H, Price SM, Shen MM, Abate-Shen C (2001). Msx homeobox genes inhibit differentiation through upregulation of cyclin D1. Development..

[32] Lee H, Habas R, Abate-Shen C (2004). MSX1 cooperates with histone H1b for inhibition of transcription and myogenesis. Science.

[33] Wang J, Abate-Shen C (2012). The MSX1 homeoprotein recruits G9a methyltransferase to repressed target genes in myoblast cells. PLoS ONE.

[34] Wang J, Abate-Shen C (2012). Transcriptional repression by the Msx1 homeoprotein is associated with global redistribution of the H3K27me3 repressive mark to the nuclear periphery. Nucleus..

[35] Wang J, Kumar RM, Biggs VJ, Lee H, Chen Y, Kagey MH (2011). The Msx1 homeoprotein recruits polycomb to the nuclear periphery during development. Dev Cell.

[36] Xie Y, Wang D, Lan F, Wei G, Ni T, Chai R (2017). An episomal vector-based CRISPR/Cas9 system for highly efficient gene knockout in human pluripotent stem cells. Sci Rep..

[37] Girish V, Vijayalakshmi A (2004). Affordable image analysis using NIH Image/ImageJ. Indian J Cancer.

[38] Koshiba K, Kuroiwa A, Yamamoto H, Tamura K, Ide H (1998). Expression of Msx genes in regenerating and developing limbs of axolotl. J Exp Zool..

[39] Holaska JM, Rais-Bahrami S, Wilson KL (2006). Lmo7 is an Emerin-binding protein that regulates the transcription of Emerin and many other muscle-relevant genes. Hum Mol Genet.

[40] Ooshio T, Irie K, Morimoto K, Fukuhara A, Imai T, Takai Y (2004). Involvement of LMO7 in the association of two cell-cell adhesion molecules, nectin and E-cadherin, through afadin and alpha-actinin in epithelial cells. J Biol Chem..

[41] Buckingham M, Rigby PW (2014). Gene regulatory networks and transcriptional mechanisms that control myogenesis. Dev Cell.

[42] Endo T (2015). Molecular mechanisms of skeletal muscle development, regeneration, and osteogenic conversion. Bone.

[43] Odelberg SJ, Kollhoff A, Keating MT (2000). Dedifferentiation of mammalian myotubes induced by msx1. Cell.

[44] Yang Z, Liu Q, Mannix RJ, Xu X, Li H, Ma Z (2014). Mononuclear cells from dedifferentiation of mouse myotubes display remarkable regenerative capability. Stem Cells..

[45] Reis-Sobreiro M, Chen JF, Novitskaya T, You S, Morley S, Steadman K (2018). Emerin deregulation links nuclear shape instability to metastatic potential. Cancer Res.

