# Correction: Emerin anchors Msx1 and its protein partners at the nuclear periphery to inhibit myogenesis

**DOI:** 10.1186/s13578-023-01001-x

**Published:** 2023-03-11

**Authors:** Zhangjing Ma, Huiyuan Shi, Yi Shen, Huixia Li, Yu Yang, Jiange Yang, Hui Zhao, Gang Wang, Jingqiang Wang

**Affiliations:** 1 State Key Laboratory of Genetic Engineering and Collaborative Innovation Center of Genetics and Development, School of Life Sciences and Zhongshan Hospital, Fudan University, Shanghai, 200438 People’s Republic of China; 2 Zhengzhou Revogene Inc, Zhengzhou, 450000 People’s Republic of China; 3 State Key Laboratory of Cell Biology, CAS Center for Excellence in Molecular Cell Science, Institute of Biochemistry and Cell Biology, Shanghai Institutes for Biological Sciences, Chinese Academy of Sciences, Shanghai, 200031 People’s Republic of China

**Correction** **:** **Cell Biosci (2019) 9:34** **https://doi.org/10.1186/s13578-019-0296-9**

In the original version of the article, the authors wish to make the following corrections:

1. The position of DAPI panels in Fig. 4g was reversed. The authors would like to provide a revised Fig. 4g with reorganized DAPI panels.
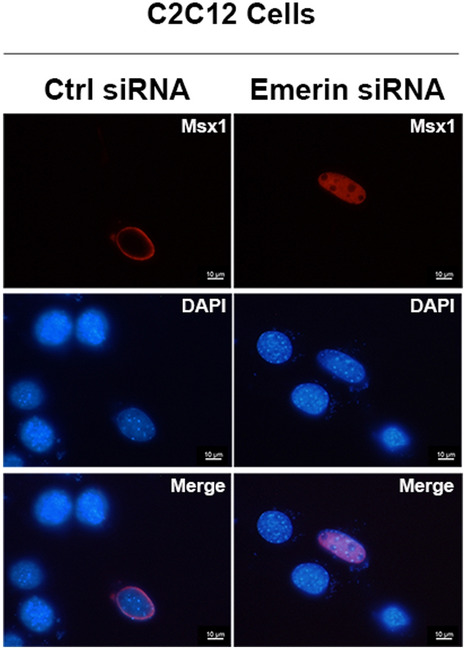
2. The Emerin blot of Fig. 2c was the unpublished data from the same Co-IP experiment published in Wang et al. [1]. The authors have repeated the Co-IP experiment and would like to provide one of the repeats to correct Fig. 2c.
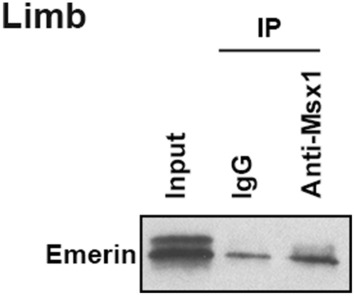

The correct version of figures is given in this correction.

## References

[1] Wang J, Kumar RM, Biggs VJ, Lee H, Chen Y, Kagey MH (2011). The Msx1 homeoprotein recruits polycomb to the nuclear periphery during development. Dev Cell.

